# Immunosuppression in Malaria: Do *Plasmodium falciparum* Parasites Hijack the Host?

**DOI:** 10.3390/pathogens10101277

**Published:** 2021-10-03

**Authors:** Carlos Lamsfus Calle, Benjamin Mordmüller, Anurag Singh

**Affiliations:** 1 Institute for Tropical Medicine, Eberhard Karls University of Tübingen, 72074 Tübingen, Germany; carlos.lamsfus-calle@uni-tuebingen.de; 2 Department of Medical Microbiology, Radboud University Medical Center, 6525 XZ Nijmegen, The Netherlands; benjamin.mordmuller@radboudumc.nl; 3 Institute for Clinical and Experimental Transfusion Medicine (IKET), University Hospital Tübingen, 72076 Tübingen, Germany

**Keywords:** malaria, *Plasmodium falciparum*, immunosuppression, tolerance, regulatory T cells, regulatory B cells, follicular T cells, dendritic cells, macrophage migration inhibitory factor, thrombospondin-related adhesive protein

## Abstract

Malaria reflects not only a state of immune activation, but also a state of general immune defect or immunosuppression, of complex etiology that can last longer than the actual episode. Inhabitants of malaria-endemic regions with lifelong exposure to the parasite show an exhausted or immune regulatory profile compared to non- or minimally exposed subjects. Several studies and experiments to identify and characterize the cause of this malaria-related immunosuppression have shown that malaria suppresses humoral and cellular responses to both homologous (*Plasmodium*) and heterologous antigens (e.g., vaccines). However, neither the underlying mechanisms nor the relative involvement of different types of immune cells in immunosuppression during malaria is well understood. Moreover, the implication of the parasite during the different stages of the modulation of immunity has not been addressed in detail. There is growing evidence of a role of immune regulators and cellular components in malaria that may lead to immunosuppression that needs further research. In this review, we summarize the current evidence on how malaria parasites may directly and indirectly induce immunosuppression and investigate the potential role of specific cell types, effector molecules and other immunoregulatory factors.

## 1. Introduction

The limited effectiveness of the control measures applied since the beginning of the century has shown that the eradication of malaria is not easy to achieve. The initial decrease in the burden of the disease has slowed down and even stopped in some countries [1] and efforts to control transmission are jeopardized due to the spread of insecticide resistance in mosquitoes [2]. Therefore, the main aim to fulfill the eradication program lies in developing an effective vaccine against *Plasmodium falciparum* (*Pf*) especially for children, pregnant women and naïve individuals who are at a higher risk of malaria-related fatalities [3,4]. So far, a considerable repertoire of vaccines targeting different stages of the parasite lifecycle have been developed, showing encouraging results in pre-clinical investigations and good vaccine efficacy in non-exposed individuals in clinical phase I trials [5,6,7,8]. However, despite the promising preliminary results in malaria-naïve humans, vaccine trials with the same vaccines and regimens in malaria-endemic populations have demonstrated a relatively lower induction of protective efficacy [9,10,11,12,13,14].

Interestingly, in endemic areas, the inability to develop a sustained protective response by a malaria vaccine is inversely associated with naturally acquired immunity against malaria infections. Throughout childhood, repeated infective mosquito bites are needed to create a tolerance to malaria symptoms and to maintain antibodies against circulating *Pf* [15]. Despite being acquired promptly and being effective in preventing complications and death, naturally acquired immunity is only partially effective and tends to be short-lived if exposure ceases. This leads to a balance between controlling immune overactivation, which may otherwise result in disease complications, and parasite multiplication [16,17,18]. Downregulation of the inflammatory response upon the repeated encounters with the parasite ensues, and symptoms occur only if a higher threshold of parasitemia is reached during subsequent infections [19,20]. This tolerance to the parasite is not exclusive to children. Unexposed adults, irrespective of their genetic background [20] also acquire tolerance to clinical malaria quickly after an initial infection [21].

**Figure 1:**
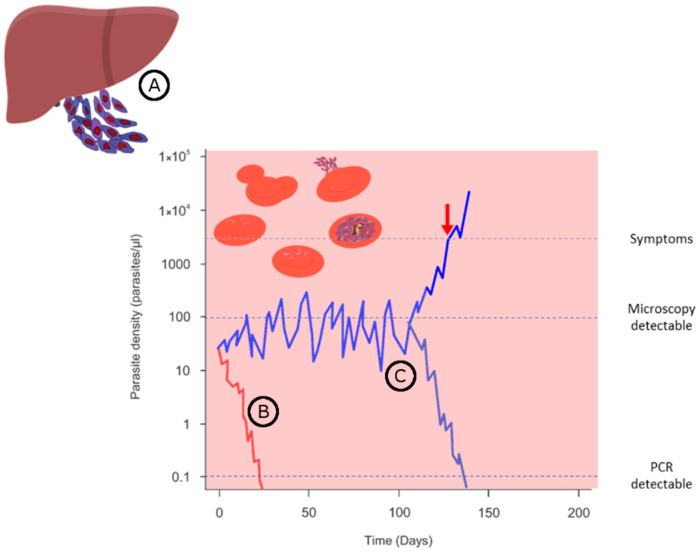
Different profiles of protection in parasite growth control seen in *Pf* endemic areas, namely: (**A**) Sterile protection at the liver stage, thus, completely asymptomatic; (**B**) Blood stage protection in which the parasite is eliminated after reaching the circulation, thereby controlling the development of symptoms; (**C**) Asymptomatic carriers control the parasite burden in the blood and remain mostly asymptomatic, eventually becoming symptomatic (red arrow) or controlling the infection.

Hence, naturally acquired immunity must develop gradually from the prevention of symptoms to full parasite control following repetitive infections over long periods. Considering this process, protection against *Pf* can be subcategorized into three major subtypes related to the variables parasitemia control and symptom development (Figure 1). Sterile protection to infection means full eradication of the parasites (e.g., in the liver), while the host remains completely asymptomatic. In case parasites are eliminated after reaching the blood, it is considered as blood-stage protection. However, the majority become asymptomatic carriers that limit the parasite burden along with malaria symptoms. Those carriers can either eventually manage to eliminate the parasite successfully or, if parasites grow over a certain threshold, symptoms may occur [22,23,24]. Sterile protection being rare indicates that naturally acquired anti-malarial immunity is skewed towards the tolerability of some presence of the parasites rather than their eradication.

The concept of tolerance during an infection can be defined as a mechanism that protects the host by reducing the negative impact of infection without, unlike resistance to infection, directly suppressing the pathogen burden [25]. This may be achieved by minimizing the damage caused directly by the parasite, its growth, or by interfering with the host immune responses to avoid a possible immunopathology created by the infection [26].

Recurring and life-threatening infections due to the dysregulation of the immune system can involve several factors at different levels of immunity [27]. Malaria-associated immunosuppression has been reported several times in the literature and has been studied for a long time. This immunosuppression could generally be defined as a reduction in the activation or efficacy of the immune system. However, due to fragmented research on its etiology [28,29,30], it became a dogma with the real mechanisms remaining undeciphered so far.

In this review, we compile and discuss different parasite components involved in promoting immunosuppression and immune regulatory factors in the host known to be affected during the infection. We performed an in-depth systematic search for relevant published work related to malaria and immunosuppression in several databases, namely PUBMED, which comprises MEDLINE, life science journals, and online books. The information gathered here may help in optimizing immunization approaches in malaria endemic populations for better acquisition of protective immunity.

## 2. Basic Knowledge on Malaria-Related Immunosuppression

Almost as old as the concept of tolerance to malaria infections [31] is the concept of immunosuppression by malaria parasites, which was postulated after the observation of coincidental paratyphoid C fever during the malaria outbreaks in British Guiana in 1929 [32].

Epidemiological evidence of immunosuppression in relation to *Plasmodium* spp. infections were noted from different observations, starting with the perception that the incidence of rheumatoid arthritis and other autoimmune processes are less frequent in people exposed to malaria compared to people sharing a similar genetic background [33]. Tolerance to malaria was observed to be a feature to inhabitants of *Pf* endemic areas and not only dependent on ethnicity [20,34]. This tolerance disappears without exposure to the parasite, which results in an overactivated immune system, symptomatology and complications upon new infections [20,35,36,37]. Moreover, as seen during several studies, children and adults whose exposure to malaria is severely reduced by relocation to a geographical area with no or very low endemicity or by chemoprophylaxis, responded better to specific antigens, resolving in better maintenance and prolonged immune memory response, than those under constant exposure to the parasite [34,38,39]. These studies provide evidence of a reduced immune response associated with the level of malaria endemicity.

Although several other parasitic and infectious diseases in malaria-endemic countries are known to immunosuppress the host [40], experiments and clinical trials in humans determined *Plasmodium* spp. as one of the main causes of immune response suppression [37,41,42,43,44]. Many other diseases and malnutrition increase the suppressive status and put the individuals at a higher risk of suffering complications [45,46]. The immunosuppressive effect by *Pf* is independent and not dependent on concomitant malnutrition or helminths [43]. Even though fever could be classified as “immunosuppressive” (due to the opposing responses that are activated to control fever), by using other febrile illnesses as control or by using asymptomatic carriers, it was possible to confirm that *Plasmodium* spp. is responsible for the observed suppression [47,48,49].

Suppressing immune responses in the host can have beneficial and detrimental outcomes depending on the intensity, timing, and duration of the suppression and the immune status at inoculation. In particular, the time of immunosuppression in the course of the disease plays an important role [50]. A suppression of immune responses is physiological and beneficial, particularly after the pathogen is cleared. Here, it downregulates the activated immune response, but it can be detrimental when present before or during infection, as it would promote immune evasion by the microorganism and can interfere with the development of effective naturally acquired immunity [51,52]. In the case of malaria, immunosuppression is primarily present during the blood stage and not at pre-erythrocytic (i.e., hepatic) stages [53]. A minimum of 4 days of blood stage multiplication are required before an immunosuppressive environment is established in the host [54]. This malaria blood stage mediated suppression is long-lasting, and extends 2–4 weeks after cure (up to 8 weeks in severe disease) [37,38,47,55,56,57,58] leaving the human host more vulnerable to reinfection, especially children [59,60,61].

In general, during an immunosuppressive reaction the effect on the immune system may be imbalanced. In malaria, there are reports during or shortly after infection of altered cellular and humoral responses to vaccines and antigens of different origin [43,56,62,63].

The human malaria blood stage can impair not only the establishment of an adequate immune response, but also previously acquired responses. Indeed, the evidence that *Pf* negatively affects the development of antibodies against an unrelated polysaccharide antigen is quite robust, whereas the evidence for protein-based antigens seems weaker [38]. Suppression of the antibody responses to protein-based vaccines is controversial and needs systematic investigation since reports are frequently incidental findings. In those studies, the primary endpoint was not designed accordingly and did not discriminate against the presence of the parasite in asymptomatic cases or in malaria. It is noteworthy that most evidence is based on antibody titers, ignoring other important aspects such as antibody avidity, affinity and subclass, or the generation of cellular memory.

Independent of the humoral affection, several others have shown a marked alteration of cellular responses during malaria, often measured as a reduction in T cell proliferation during stimulation [37,43,55,56,57,64,65,66]. The adaptive immunity is compromised at several levels during the memory generation and its maintenance.

## 3. Host Regulatory Cells in Malaria Infections

**Figure 2:**
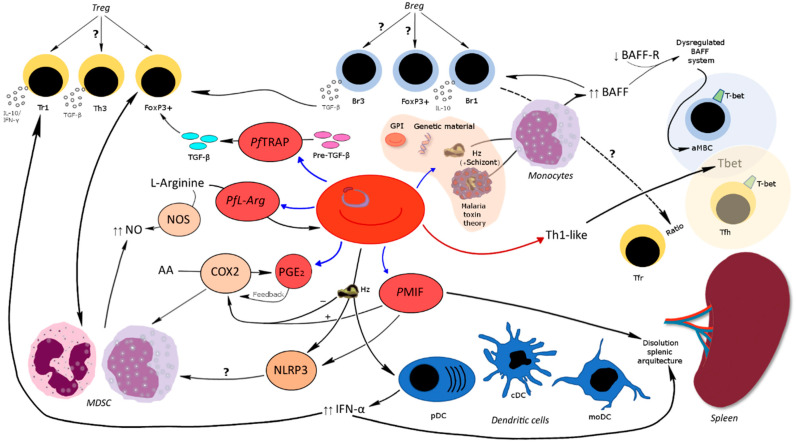
Summary of immune system alterations related to *Pf*. The parasite in the center can produce a variety of components during its development (blue arrows) or promote a response (red arrow) that has the potential to interfere with cellular and humoral immunity. In the red bubbles the components produced by the parasite described in the manuscript and in shadow next to the parasite are the components implicated in the malaria toxin theory. In italics: immune system components proposed during malaria infection that could contribute to immunosuppression in the host. Black arrows connect the different immune processes described in this manuscript. The blue shaded bubble and the yellow shaded bubble represent the B-cell compartment and the T-cell compartment in the secondary lymphoid organs, respectively. Dashed arrow connects the possible relationship of Breg with the Tfh/Tfr balance. GPI—Glycosylphosphatidylinositol; Hz—Hemozoin. “**?**” reflects possible but unexplored pathways.

Host lymphocytic and myeloid regulatory cells maintain homeostasis to immune responses, and help to overcome inflammation after resolving the infection and avoid further host damage. However, in some infections, the very same regulation is jeopardized, causing disease or lack of proper immune responses [67]. These regulatory cells modulate the immune response from the level of the antigen-presenting cells to the generation of effector cells and antibodies. There is some evidence that the main regulatory cellular populations of T and B lymphocytes as well as myeloid cells play a crucial role during malaria pathogenesis and disease outcome (Figure 2).

### 3.1. CD8^+^ Regulatory T Cells

Initially, it was thought that the main regulatory T cells implicated during malaria were the CD8^+^ suppressor cells [42,65] since the removal of all CD8 T cells contributed to higher in vitro lymphoproliferation to different stimuli. However, the fact that no increase in immunosuppressive Leu-2 T cells was observed during human malaria and that their depletion by complement did not contribute to the restoration of T cell proliferation contradicted this hypothesis. Their role in immunosuppression might be minimal, but this has not been further investigated. Independent of these CD8 regulatory T cells, there are CD4^+^ regulatory cells able to suppress not only antigen-specific CD4 responses, but also the CD8 responses [68,69,70].

### 3.2. CD4^+^ Regulatory T Cells

Regulatory CD4^+^ T cells (Treg) are a heterogeneous population which modulate the immune system primarily by suppressing the proliferation of effector T cells. The most studied type of Treg is FoxP3 expressing Treg, which originates either as naturally occurring Treg derived from the thymus or as CD4^+^ converted into Treg by FoxP3 expression. However, there are other types of inducible Treg that do not express FoxP3, which can be divided mainly in IL10/IFNγ-producing Th1-like (Tr1) cells and TGFβ-producing CD4^+^ (Th3) cells [71,72,73,74].

*Pf* infection triggers regulatory T cells as seen in a co-culture experiment of PBMCs from healthy donors with infected RBCs (iRBCs) where the expression of FoxP3 in CD4^+^ cells is upregulated through the help of activated monocytes [75]. Indeed, the expansion of FoxP3^+^ Treg can be detected in the early phase of the *Pf* blood stage infection [73], subsequent to a TGFβ peak in the circulation. However, not only TGFβ is needed for their proliferation but also IL2 and IL10 [75]. It is noteworthy that apart from being secreted by monocytes, TGFβ can be actively converted by the parasites from its latent form by a thrombospondin-related adhesive protein (TRAP) [76,77,78]. The parasites, using their TRAP human ortholog (*Pf*TRAP), may actively induce suppression by TGFβ induced FoxP3 expression in T cells [77,79]. The key involvement of this cytokine is evident when anti-TGFβ prevents Treg immunosuppression [80,81]. During controlled human malaria infections (CHMI), TGFβ is known to precede pro-inflammatory cytokines [82,83] suggesting parasites may activate TGFβ to dampen initial pro-inflammatory responses [73]. This activation seems to be sufficient at low parasitemia since even submicroscopic *Pf* blood stage inoculum in CHMI produces changes in the CD4^+^ T cell phenotypes [84]. Interestingly, Fulani people, known for their low susceptibility to *Pf* malaria infections, present lower Treg and TGFβ levels [85]. All this suggests that already in an early exposure to parasites, mechanisms of suppression are activated.

In malaria-endemic areas, Treg expressing FoxP3 are correlated with higher parasitemia and lower pro-inflammatory responses [73] and lower numbers are associated with better disease outcome [63]. Torres et al. suggested the importance of considering all alternative types of regulatory T cells in immune suppression during malaria as FoxP3^+^ Treg numbers or proportions were found unrelated to the disease symptoms [86]. Albeit that FoxP3 expressing T cells do not differ in numbers during uncomplicated or severe malaria cases, Tr1 co-producing IL10 and IFNγ were reported to be higher during uncomplicated malaria compared to severe malarial cases [87] where FoxP3^+^ T cells with a potent suppressor capacity, recognized by TNFR2 expression, were more common [88,89]. Indeed, despite heavily exposed children showing a reduced frequency of FoxP3 and TNFR2 expression [90], CD4^+^ T cells expressing IL10 were more prominent, while the CD4 response in adults was dominated by IFNγ and TNFα production [91]. In contrast to the protection against clinical malaria associated with this type of IL10-producing CD4^+^ T cells, protection against infection was governed by a different CD4 response profile, but both types of protection are considered part of the naturally acquired immunity against malaria [92]. The responses of each CD4 cell should be analyzed simultaneously to discern their contribution to host survival and to assess whether the benefit of clinical protection outweighs the risks of immune evasion of the parasite. This reflects the importance of testing different types of suppressor cells to assess each type’s contribution to progression of the infection.

Initially, the idea that pre-existing Treg might influence the susceptibility to following malaria infections was postulated based solely on CD25^hi^ Tregs measurements from a single study until Portugal et al. provided supportive information [73]. Following infection, there is a shift towards lower pro-inflammatory reaction by higher levels of IL10 and TGFβ, and an increase in FoxP3 negative Treg co-expressing IL10, IFNγ, and TNF [93]. This profile remains only under constant exposure and is common in children carrying asymptomatic parasitemia during the dry season, suggesting that inflammation is finely tuned to control the parasite and not produce symptoms [93,94].

Regarding the survival component conferred by the profile of Tregs under persistent contact with the parasite, in a study in children 6 to 24 months of age in which they strongly adhered to chemoprevention, a greater number of protection-related CD4^+^ cells (IL2 and TNFα) than IL10-producing CD4^+^ cells developed. This profile corresponded to 55% fewer infections and episodes of malaria compared to placebos during follow-up [95]. The long antimalarial effect conferred using dihydroartemisinin-piperaquine in those children preventing further malaria may serve as an additional sign that if exposure to parasite disappears, immunity is better acquired.

Chronic (or repetitive) exposure to malaria is reflected on T cells as long-lasting perturbations known as “exhaustion markers”, such as the aforementioned TNFR2 [96]. Over the last decade, the understanding of key steps in the regulation of T cell responses has led to the discovery of what has been named as “immune checkpoints” [97]. These, known in malaria as “exhaustion markers”, are surface proteins in malaria pathogenesis that play a role in immune mechanisms to promote or impede protection through co-stimulatory or co-inhibitory receptors, respectively [96]. The expression of “exhaustion markers” on CD4^+^ cells, together with the presence of Tregs and anti-inflammatory cytokine production, are long lasting effects in children even after cure, which may contribute to a prolonged state of immunosuppression until the next infection [98]. These “immune checkpoint” pathways controlling tolerance and tissue collateral damage during malaria infections have recently been reviewed elsewhere [96].

The immune memory against *Pf* is difficult to develop and is lost quickly. When some of these “immune checkpoint” pathways are expressed on CD4^+^ effector memory T cells, they can become, through co-expression of IFNγ and IL10, suppressive cells for other CD4^+^ T cells [99]. Finally, these cells, switching from memory T cells to suppressive cells, will undergo apoptosis [100], which may contribute to a shrinking memory compartment in the body. An additional mechanism to reduce the memory compartment is that Tregs can contribute to depriving other T cells of IL2 by displaying a higher affinity for this cytokine than the non-regulatory ones, triggering apoptosis by deprivation in the last ones [72].

While the presence of Treg in the general population may contribute to clinical tolerance conferring a survival component, it is deleterious for the generation of cellular memory, diminishing a long-term immune memory response against the parasites.

### 3.3. Regulatory B Cells

Similar to Tregs, Bregs are part of physiological mechanisms to balance the immune system, avoiding autoimmunity or excessive inflammatory responses during and after infection [101]. Bregs are also known to be involved in potentially harmful suppression of humoral and cellular responses during bacterial, viral, and parasitic infections [102]. Breg activation by pathogens can be driven through TLR and amplified by CD40 and B cell receptor (BCR) signals [103]. Bregs can be divided into Bregs producing IL10 (Br1), Bregs producing TGFß (Br3) and FoxP3 expressing Bregs (BFoxP3) [104]. The most studied ones in malaria are Br1, which include all B cells producing IL10 [105,106]. Br1 are mostly found in the spleen and the peritoneal cavity, but a small proportion of them circulates in the blood together with an accessory B cell population with the potential capacity to become Br1 [104]. IL10 produced by Br1 cells creates a positive feedback loop increasing IL10 Bregs. In contrast, in other Bregs, such as Br3, despite promoting Treg by TGFβ production, the same cytokine drives Br3 apoptosis, self-controlling Br3 proliferation [104]. In fact, B cells are suggested to be as important as T cells in IL10 production, as seen in murine malaria [105].

A recent murine model showed an increase in splenic Bregs from day 5 postinfection during uncomplicated malaria [105]. The adoptive transfer of the very same cells during infection resulted in a transient increase in parasitemia and the suppression of Th1 responses, which would favor the growth of the parasites without causing death to the host [105]. This would indicate the importance regarding the timing of IL10 Breg generation. Despite their involvement in protection from cerebral malaria [107], Han et al. postulated that the activation of Bregs by the parasites might be implicated in the reduced intensity of secondary antibody responses [105]. Therefore, not only the timing of Breg expansion is important, but also the interaction in germinal centers (GC) between different T cells and Bregs for B cell memory generation [62].

Although evidence is limited [105,106], involvement of Bregs during acute human malaria seems likely since there is evidence of increased circulatory “B cell-activating factor belonging to the TNF family” (BAFF) during uncomplicated malaria [106]. Bregs are supported by BAFF treatment in vivo or in vitro by promoting the survival of IL10 producing B cells [108,109,110].

#### 3.3.1. BAFF System Molecules

“BAFF system molecules” include two ligands (BAFF and a proliferation-inducing ligand (APRIL)) expressed mainly by myeloid cells and three receptors (BAFF receptor (BAFF-R), transmembrane activator and calcium modulator and cyclophilin ligand interactor (TACI) and B cell maturation antigen (BCMA)) expressed in most B cell lineages, where they promote the survival, activation, proliferation, and differentiation of B cells, typically in the spleen [111]. Recently, Dechkhajorn et al. observed in human lymphoid tissues from patients with fatal malaria that parasitemia is associated with changes in the BAFF molecules system [112].

Some pathogens are known to take advantage of BAFF system molecules to drive the humoral immune response to their benefit by increasing BAFF ligand expression and suppressing the expression of TACI and BAFF-R receptors [111]. Indeed, BAFF levels are found to increase during *Pf* malaria in naïve or previously exposed individuals [112] as well as to be upregulated in placentas after placental malaria [113]. Even neonates in a malaria endemic country showed higher BAFF in cord blood compared to neonates in a Western country [114]. This increase in BAFF levels can be influenced in macrophages by IL10 or IFNγ [115]. In vitro, hemozoin (Hz) and the soluble antigens of the schizont stage can stimulate BAFF secretion by monocytes and BAFF-R expression in B cells [111]. In vivo, however, the expression of BAFF-R is rather low during the acute phase of human malaria [111]. Since this receptor promotes B cell survival, the lower expression BAFF-R could explain why there would be insufficient memory B cell formation after natural infection [111]. Accordingly, children expressing more BAFF-R on B cells are those with the highest plasmatic levels of parasite-specific IgG and IgM [116,117]. In contrast to acute malaria [118], BAFF-R was strongly expressed on B cells in the spleen and lymph nodes in autopsy samples from patients who had died from severe malaria [112]. However, when compared to the autopsy samples of severe trauma patients, mRNA expression of BAFF-R was significantly reduced in the lymphoid tissues [112].

Despite being more highly expressed during infection, BAFF is not able to support antigen-secreting cell (ASC) survival by binding to TACI and BCMA receptors in pediatric malaria [111], since the form of BAFF found in circulating serum is not its ligand [118,119]. Additionally, BAFF levels have also been negatively associated with *Pf*-specific and total IgG^+^ subsets of memory B cells in infants [120]. By contrast, APRIL, which is over BAFF the main ligand of those two receptors, is highly produced in *Pf* infections [121]. With the above in mind, malaria shows a potential to dysregulate the BAFF system, which could potentially contribute to disease chronicity [119].

#### 3.3.2. Atypical Memory B Cells

An increase in plasmatic BAFF and a decrease in BAFF-R has been proposed to promote atypical memory B cells (aMBCs) at the cost of classical memory B cells (MBCs) development [117]. Indeed, BAFF plasma levels correlate with the proliferation in the body of aMBCs [118], with both being linked to the cumulative duration and frequency of parasite exposure [44,62,122,123,124]. The direct capacity of parasites to interact with B cells became patent in in vitro co-cultures of *Pf* strains with B cells [125]. Here, it was shown that their contact-dependent interaction promoted and enhanced parasite growth along with an increased proportion of atypical memory B cells and a reduction of the classical type [125]. This pathogen-driven aMBC development can reach 50% of the total circulating B cells in malaria endemic exposed individuals, while in healthy non-exposed individuals the percentage is around 5% [123]. These types of B cells are also commonly observed in other chronic infections [126] and resemble an “exhausted” cell phenotypic appearance with a decreased capacity to differentiate into ASCs and secrete antibodies [108]. When humans move to low transmission settings or to areas of total absence of parasite exposure, a decline in aMBCs is observed, consistent with the fact that long-lived *Plasmodium*-specific MBCs are retained in these individuals even without a frequent booster [62,122,124,127,128]. Malarial dysfunctional humoral response is therefore linked to the presence of aMBCs [62].

aMBCs are a heterogeneous population of B cells, but share common features [123] such as increased inhibitory receptors and chemokine receptors, reduced expression of costimulatory molecules, and are hyporesponsive to BCR stimulation [123]. Despite missing phenotypic markers of memory and showing anergic characteristics in response to BCR signaling, they are able to secrete antibodies and proliferate under IL2 and IL10 cytokine stimuli [123,126]. *Plasmodium*-specific aMBCs have been shown to proliferate in the body at similar frequencies as the classical MBCs, but have a shorter life span [62,108,128,129], which generates shorter specific responses against the parasite, maintained only under its presence [108,128]. This short effect is supported by the observation that in CHMI in malaria-naïve individuals, aMBCs proliferate promptly throughout the course of the infection, suggesting that they do not originate from the GC and thus do not follow memory generation [118].

Tbet, being one of the most characteristic phenotypic markers expressed on aMBCs, is driven by *Pf* malaria either by antigen exposure or by the immune reactions triggered by the parasite [123]. Either way, expression of Tbet in naïve B cells is commonly induced by IFNγ through a feed forward mechanism [108,130,131] and IFNγ plasma levels are also correlated positively with the increase in aMBCs early in infection [118]. Additionally, IgG3, which is expressed on Tbet expressing aMBCs, is the most common IgG subtype found in acute malaria serum samples and correlates with IFNγ levels as well [123]. Interestingly, the antibody repertoire generated from aMBC populations differs from that of classical memory B cells [129]. The parasite can modify the MBCs compartment [122] and also overexpress BAFF [111], which promotes nonspecific differentiation of B cells into ASCs. The splenomegaly observed in patients with malaria has led some to suggest it to be linked to hypergammaglobulinemia [108].

Based on current knowledge, the involvement of aMBCs in immunosuppression during malaria is not definitive, but their importance in chronic infections and in autoimmune disorders makes a contribution to this phenomenon plausible [62].

### 3.4. Follicular Regulatory T Cells and Follicular Helper T Cells

Chronic exposure to *Plasmodium* is associated with similar phenotypes in both B cells and T cells [44], which are characterized by the expression of “immune checkpoints” (such as CTL-4) and Tbet. For the generation of long-lived memory by plasma cells and MBCs, the interaction of follicular helper T cells (Tfh) and B cells in the GC of lymphoid organs such as the spleen is necessary [62,132].

The cytokine environment created during blood stage malaria polarizes the T cell response towards Th1-driven immune responses that affects the expression of Tbet on B cells [123] as well as on Tfh cells [133,134]. A recent study showed that when Tbet is expressed on B cells of the GC then antibodies with higher avidity and affinity are generated, inducing a more effective immune response [135]. In contrast, Tbet expression in Tfh prevents their progression into mature Tfh, reducing the overall magnitude of the GC response [108,124]. Indeed, Chan et al. have recently suggested that in humans the Th1 profile of circulating Tfh in malaria infections are associated with a disruption of GC formation and with the induction of aMBCs, thus affecting long-lived and memory humoral responses [136]. The plasma cells originating from this Th1 response have previously been shown not to contribute to long-lasting humoral immunity, but to the opposite [137]. This suggests that over repetitive infections more effective, but short-lived antibody responses are generated, which would result in slow acquisition of protection.

Tbet expression on Tfh leads to the accumulation of less functional immature Tfh [138], disturbing the splenic architecture and hampering the interaction with B cells [124,139,140]. This becomes evident when IFNγ or Tbet expression is blocked, restoring Tfh functionality [108,130,135]. For example, in mice, *Plasmodium* macrophage migration inhibitory factor (*P*MIF) upregulates IFNγ and IL12, which contributes to Tbet expression and to the Th1 proinflammatory response. This may inhibit Tfh maturation in humans and induce improper GC formation and B cell response [141]. Although Tfh are crucial in the CG for an effective humoral response in primary and secondary infections [124,142], they apparently undergo plasmodium-induced migration out of the CG that favors a short-lived antibody response [138].

It has been hypothesized that, notwithstanding the contribution of any other possible immune interference promoted by or for the parasite during the blood stage, becoming clinically tolerant promotes functional Tfh responses due to decreased levels of pro-inflammatory cytokines when reinfected [122].

In addition to Tfh, follicular regulatory T cells (Tfr) exist. They are expressing Tfh markers, but contrary to Tfh, which stem from naïve CD4^+^ T cells, Tfr have a closer relationship to Treg and also express FoxP3 [143,144]. The expression of the chemokine receptor CXCR5 enables them to access and accumulate in B cell lymphoid follicles of the GC [143] and become Tfr by the downregulation of CD25 expression [143]. These Tfr have the potential to inhibit Tfh proliferation and maturation, thereby distorting the GC reactions. Their accumulation in lymphoid organs in other chronic diseases has already been described [143] and their presence in the circulation has been observed during malaria [145,146]. In humans, Tfh and Tfr cells are mostly investigated in peripheral blood as a surrogate since access to lymphoid organs is limited [122,138,143].

The ratio Tfr:Tfh, more than the frequency of Tfr, and the expression of CTLA-4 seems to drive the suppression seen in chronic infections [143], and influences the length of responses of MBCs and plasma cells in the GC [124]. The kinetics of Tfr in febrile malaria in children are of significant interest where Tfr expresses different “immune checkpoints” that are important for their mechanism [143,145]. It has been suggested that Tfr expressing low CTLA-4 enhance the proliferation of IL10 producing Bregs [143]. Interestingly, it is known that when CTLA-4 is depleted in Treg there is an increase in IL10, which may contribute further to increased GC responses [147]. Despite all the reported results, the overall role of IL10 in the axis of Tfh and GC interactions remains controversial [143,147].

Bregs have been linked to the regulation of Tfh/Tfr balance [148]. Deregulation of this equilibrium can contribute to the generation of autoantibodies [149]. Persistent infections, such as malaria, can promote the generation of antibodies recognizing endogenous epitopes [150]. These medium- or low-affinity antibodies are polyreactive, recognizing *Pf* and human antigens simultaneously [151]. The generation of autoantibodies has been associated with malaria, not only due to persistent infection, but to *Pf* extracellular vesicles as well as to various parasite molecules, which mimic host antigens [152,153,154,155,156]. This reflects the weakness of the tolerance mechanism to self-antigens in the host during malaria infection.

The suppression conferred by Tfr in GC on B cells and Tfh is durable, but reverts with IL21 [143]. While IL 21 regulates the proper maturation of Tfh and limits the development of Tfr [144,157,158], Tfr cells repress the production of IL21 [159]. Despite the described increases in this cytokine in *Pf* infected humans [160], the dual expression of IFNγ and IL21 by Tfh Th1-like promotes lower B cell activation, triggering less efficient B cell responses which are commonly seen in HIV, tuberculosis, and malaria infections [133]. IL21 is critical to create parasite-specific B cell responses [139,159] and disrupting IL21 signaling impairs protective humoral immunity to malaria [144,161]. Therefore, IL21, as a pleiotropic cytokine, has even been suggested as an adjuvant for vaccines to induce better immunity acquisition against the parasite [160], although this idea should be taken with caution [144].

To summarize, the contribution of the lower maturation of Tfh to modifying B cell populations, as well as the generation of Treg or Tfr in lymphoid organs should not be dismissed [108,162], nor should the contribution of B cell types in modifying T cell responses be ignored [132,163].

### 3.5. Dendritic Cells 

Myeloid cells are key players in the defense against infectious diseases including malaria, and they orchestrate the development of antimicrobial immunity, regulating the innate and adaptive immune systems. Dendritic cells (DC) are essential and highly effective in recognizing and presenting heterologous antigens. When exposed to malaria parasites, downregulation of several of their functions can be observed [164].

DCs are a heterogeneous cell population, characterized by a variety of phenotypic markers, and reside in different compartments of the body, to survey and maintain tissue homeostasis [165]. Based on their development from different cell lineages, circulating DCs are categorized as conventional DCs (cDC) and plasmacytoid DCs (pDC), if they originated from myeloid or lymphoid precursors, respectively [165]. Changes in cDC and pDC can be found in the peripheral circulation of malaria-exposed individuals. Nevertheless, the most studied type of DCs are the monocyte-derived DCs (moDC), which are derived from monocytes by in vitro differentiation [164]. The suppression of moDC when co-cultured with parasites has been found to be dependent on the culture ratios. While low DC:parasite ratios activate DCs, high DC:parasite ratios suppress their function [166,167]. The limitation of this approach is the usage of different parasite components and strains as stimulants, with variations in interlaboratory protocols, which lead to contradictory findings [164,168]. Moreover, it was recently reported that laboratory generated moDCs can exhibit indistinguishable pro- and anti-inflammatory characteristics with large interdonor variation [169], making them less suitable for experimentation on DC immunosuppression in malaria. The moDCs do not represent the phenotypical and functional characteristics of the circulating DC types in malaria [165,170], but instead they might be a closer representation of DC types located in spleen or in other non-lymphoid organs linked to pathology [171]. Therefore, when feasible, the use of DCs isolated from the infected host might represent a better approach to resolve the direct involvement of *Plasmodium* spp. on the DC immunosuppression.

In high transmission settings, individuals under current or recent malaria infection show a decreased number of DCs in the circulation, with an exception of variation in some cDC subtypes. However, whether these variations in the circulation reflect a relocation of the cells or immunosuppression is not yet known [164]. A distortion seems likely since these blood changes are accompanied by an increase in DC apoptosis, a reduction in DC maturation, lower cytokine secretion, and reduced phagocytosis with a reduced capacity to induce T cell proliferation impeding a proper DC function [58,172,173,174,175,176,177]. This malaria-mediated DC impairment is less pronounced in lower transmission areas, suggesting DC functionality being restored along with a reduction in malaria prevalence [173,178,179,180].

Nevertheless, regardless of whether malaria is mild or severe, or whether individuals are previously naïve or naturally exposed, DC phenotypes do not reflect major differences once malaria-induced dysfunction has been established [164]. The systemic inflammation during symptomatic infection triggered by a certain threshold of parasite biomass seems to be a determinant for DC impairment, which is prolonged for days after curative treatment [58,172,174,181]. In CHMI, while cDC recovered quickly to the levels previous to infection, pDC remained at 47% for 60 h post cure [172].

The DCs during asymptomatic and submicroscopic infection might still contribute to modulating immune responses by the parasite [164,174,182]. DNA bound to uric acid or Hz activates downstream pathways of the TLR9 expressed only on pDC, which further reverts in IFNα production [164,174]. The pDC are the major source of this type I interferon during *Plasmodium* infection, with an identified role in impeding proper humoral immunity against the parasite [183,184] and reducing fatality rate [185]. Type I interferon is a recognized family of cytokines relevant for their involvement over several infections in protecting against the pathogen, but alongside producing immunosuppression, which ends in microorganism survival instead [182,186]. This was confirmed by the IFN type I blockade, resulting in a better production of parasite-specific antibodies that controlled the parasitemia more effectively, alongside a greater spleen structure and improved T cell responses [162,185]. Moreover, the generation of Type I interferon was established to be at an early time point of blood stage infection [175,182,187], suggesting a possible active participation of the parasite in promoting their survival.

Heretofore, the influence of *Pf* on DC functionality is not clearly understood, also due to variation in the study designs, the phenotypic markers analyzed and the fact that the main parasite stage tested has been the blood stage [164] until recently [181]. There is growing evidence that DC suppression in the blood is due to the intracellular blood stage and not merozoites. The latter is instead driven by low co-stimulatory marker expression on DC and high production of cytokines [181]. Merozoites able to sustain a potent activation of DC were not found to be efficient at rescuing the suppression conferred by the infected erythrocytes [181], which was dependent on the proportion of parasites to DC and independent of the surface components of the infected cell [181]. Yap et al. propose three ways to recognize impaired DC function in malaria: define the phenotypic profile of DC suppression, discover how malaria parasites influence the DC activation, and investigate whether this interaction caused is by cell to cell contact or by other indirect mediators [164].

### 3.6. Myeloid Regulatory Cells and Myeloid-Derived Suppressor Cells

More recently, a myeloid regulatory cell lineage composed of polymorphonuclear leukocytes, macrophages, DC, and myeloid-derived suppressor cells (MDSCs) that seems to be important for downregulation of the immune system has been systematically characterized [188]. These cells, with potent regulatory properties, physiologically exert immune suppression and tolerance on both the innate and adaptive immune systems. When triggered by pathogens or self-antigens, they contribute to the generation of a tolerogenic microenvironment and favor the generation of Tregs [188].

Specifically, MDSC, a well-studied group of cells supporting cancer cell growth [189,190] seem to play an additional role in the pathophysiology of infectious diseases [40,191,192,193,194]. Parasitic infections can promote the expansion of this type of myeloid cells in the host, thereby dampening antigen-specific T cell responses, and a role in malaria has been suggested [40,195]. Recently, we reported an expansion of MDSC in the circulation of malaria-naïve human individuals undergoing CHMI [196]. Due to their heterogeneous phenotype and immature myeloid appearance, they have not been described well in malaria pathogenesis till now. During infections, several immune cells are recruited from the bone marrow compartment to the spleen, playing a key role in the course of the pathogenesis. In response to malaria infections, there is a decrease in early myeloid cell progenitors in the bone marrow and a portion of these cells are mobilized to the spleen, expanding the myeloid niche in this organ [197,198].

While direct evidence of MDSC involvement in the pathophysiology of malaria is scarce, several effector molecules involved in MDSC-mediated immunomodulation are also known to be increased during malaria; e.g., it has been shown that PGE2 (reported to be produced by the parasite [199]) and COX2 can induce conversion into MDSC instead of DC [200]. Short exposure to PGE2 promotes maturation of DC, but prolonged exposure drives differentiation towards MDSC [201]. As an additional hint, the mechanism of MDSC-mediated suppression is in part promoted by nitric oxide (NO) production, a well-studied molecule in malaria [202]. Deprivation of L-arginine, a substrate of the enzymatic reaction producing NO, leads to suppression of T-cell proliferation by arresting their division cycle [203]. The parasite possesses an enzyme (L-arginase) that may contribute to further deprivation of the body of L-arginine [204,205,206]. NO produced by MDSCs has also been shown to inhibit the antigen presentation of DC to CD4 T cells [207]. Another mechanism that has been shown to induce MDSC generation is the activation of inflammasomes such as NLRP3 [208]. Interestingly, it has been reported that the inflammasome can be stimulated by the parasite-derived Hz. When monocytes and other phagocytic cells phagocytize Hz, their activity is altered and ROS and HNE production increase, triggering innate immunity by toll-like receptors (TLR) and NLRP3 and AIM2 inflammasome [209,210]. This phenomenon might stimulate MDSC expansion in the spleen, as reflected by IFNγ and TNFα-induced nitric oxide synthase (NOS) expression. Moreover, all plasmodium species produce their own macrophage migration inhibitory factor (MIF) and, as seen, an inhibitor of MIF reduces NLRP3-dependent IL1β released in response to malaria parasite infection [141,209,211,212]. IL1β has a pivotal role in cancer as well as in MDSC development [213,214]. Of note, human MIF (huMIF) induces tumor growth and metastasis by inducing MDSC in the tumor [215], which promotes immunosuppression in the tumor’s microenvironment [216].

In conclusion, there is ample evidence that *Plasmodium* spp. modulate the host myeloid immune responses not only to generate inflammatory responses, but also by inducing immuno-suppressive reactions.

## 4. Concluding Remarks

*Plasmodium falciparum* parasites regulate the immune system by active direct or indirect mechanisms and take advantage of other triggered host processes during infection, rendering them beneficial for their own survival (Box 1).

The involvement of the parasite in the immunosuppression of the host is clearly identified during asexual development of the blood stage, which impairs the generation as well as the function of humoral and cell-mediated immunity [22,63]. Detected by different immune perspectives, malaria-related immunosuppression is reported to endure for several days after curative treatment. Interestingly, malaria starts to be perceived as a chronic infection either due to constant submicroscopic asymptomatic infections or by repeated infections over long periods of time. Repeated malaria episodes tailor the immune response [61] and the immune profile generated persists as long as the parasite remains in contact with the host. While parasite exposure vanishes or declines, *Plasmodium*-specific immunity increases. This is observed in the profile of memory B cells in unexposed individuals several years after initial contact [62,119]. Parasites and humans may have evolved together for years to find an equilibrium where there is a survival component acquired by both. Survival in humans goes through the avoidance of immunopathology by a highly activated immune system that activates tolerogenic pathways, which in turn can favor the parasite being less recognized by the immune system, evading its elimination. The naturally acquired immunity in adults robustly prevents complications and death, but does not lead to sterile resistance to infection with increasing age or exposure [217,218]. The best evidence that proper immunity is more beneficial than immune tolerance comes from the Fulani community. This ethnic group presents with lower infection rates and lower parasite densities with fewer symptomatic cases of malaria, coincidental with proper immune responses [219].

Box 1 **Basic Aspects of the Suppression of Immune Responses in Malaria.**

- Only during or shortly after infection with *Plasmodium* spp.;
- With possible exceptions (Fulani ethnic group [3,4]), it is not merely inherent to any ethnic or host phenotype;
- Genuinely caused by *Pf* and not by a concomitant infection, malnutrition, or as a response to fever;
- Prolonged effect, about 2–4 weeks (up to 8 in severe malaria) following acute infection before returning to normal;
- The immunosuppression develops in the host during the growth of the parasite or is established through continuous and repetitive infections;
- The contributions of the different stages of the parasite’s lifecycle are distinct;
- During malaria, there are plasmatic components with suppressive properties (parasite cellular components);
- Malaria affects cellular as well as humoral responses in the host, suppressing T cell proliferation and antibody production.

The malaria-immune distortion resembles, in several aspects, the one seen in these other chronic infections that coexist with *Pf* in endemic regions. Their contribution would produce a more pronounced distortion of the immunity in the host that tends towards a chronic immune-compromised status, hindering the restoration of healthiness and contributing to pathology by co-infection [61]. For further insights, it would be interesting to test a tightly controlled immunosuppression wash-out period during which participants take chemoprophylaxis against malaria and other possible contributing diseases, to test afterward the same regimens of malaria vaccines in those individuals. Indications of promising results come from Obiero et al. who compared a *Pf*-specific cellular response between Tanzanian and Dutch participants before and after CHMI. While responses were comparable at baseline, Tanzanians showed lower lymphocyte IFNγ production after CHMI and the immunosuppression, still recognizable 1 month later, was suspected to be produced by the blood stage exposure during CHMI infections [220].

Parasites generate different components that can influence host responses. These have been mentioned throughout this manuscript, such as *P*MIF or *Pf*TRAP, but there are other components that follow the bacterial endotoxinlike tolerance theory, known as “malaria toxins, “which have been reviewed elsewhere [28,30,210,221]. One of the approaches being pursued is immunization against these parasite components [222] as well as antibody blockade of “immune checkpoints”, which could be advantageous for the proper induction of immunity. Immunotherapy is booming in different infectious diseases and cancer, which could be extrapolated to human malaria [223].

Immunity to malaria develops slowly and is rapidly lost, so in order to achieve the goal of eradicating malaria by 2030, we need, alongside the development of a potentially efficacious vaccine, a healthy immune system. For that, it will be crucial to promote a robust immune system, not only by adjuvants, but also by factors restoring the capability to respond efficiently to vaccine immunization.
